# The Code of Ethics

**Published:** 1882-03

**Authors:** 


### The Code of Ethics.

In the present issue we would merely notice the following "Substitute for the report of the special committee on amendments to the system of medical ethics," offered by Dr. D. B. St. John Roosa, of New York.

"The Medical Society of the State of New York, in view of the apparent sentiment of the profession connected with it, hereby adopts the following declaration, to take the place of the formal code of ethics, which up to this time has been the standard of the profession in this State:

"With no idea of lowering, in any manner, the standard of right and honor in the relation of physicians to each other, but on the contrary in the belief that a larger amount of discretion and liberty in individual action, and the abolition of detailed and specific rules, will elevate the ethics of the profession, the medical profession of the State of New York, as here represented, hereby resolve and declare that the only ethical offenses for which they claim and promise to exercise the right of discipline, are those comprehended under the commission of acts unworthy a physician and a gentleman.

"*Resolved*, Also, that we enjoin the county societies, and other organizations in affiliation with us, that they strictly enforce the requirements of this code."

A full discussion of this proposed amendment is evidently impossible in a simple editorial note. We may, however, briefly remark that while it illustrates fully the liberal tendency of the day, the door might thereby be thrown wide open for the entrance of numberless abuses, and the result, be a perceptible lowering of the whole standard of medical ethics, already none too high.

It is generally conceded that some changes in this direction are advisable; but the policy of removing all restraint from unprincipled men who call themselves "physicians and gentlemen," is a very questionable one, to say the least.

